# Melanoma differentiation-associated gene 5 in zebrafish provoking higher interferon-promoter activity through signalling enhancing of its shorter splicing variant

**DOI:** 10.1111/imm.12179

**Published:** 2014-01-09

**Authors:** Peng Fei Zou, Ming Xian Chang, Na Na Xue, Xue Qin Liu, Jun Hua Li, Jian Ping Fu, Shan Nan Chen, Pin Nie

**Affiliations:** 1 State Key Laboratory of Freshwater Ecology and Biotechnology, Institute of Hydrobiology, Chinese Academy of SciencesWuhan, Hubei Province, China; 2 Graduate University of Chinese Academy of SciencesBeijing, China; 3 College of Fisheries, Jimei UniversityXiamen, Fujian Province, China; 4 Fisheries College, Huazhong Agricultural UniversityWuhan, Hubei Province, China

**Keywords:** melanoma differentiation-associated gene 5, mitochondrial antiviral signalling protein, splicing variant, type I interferon, zebrafish

## Abstract

Melanoma differentiation-associated gene 5 (MDA5) is one of the three members in the retinoic acid-inducible gene I-like receptor (RLR) family, which are cytoplasmic pathogen recognition receptors recognizing intracellular viruses. In the present study, MDA5 and its spliced shorter forms, named as MDA5a and MDA5b, were identified in zebrafish. MDA5a and MDA5b can be up-regulated in cell lines following the infection of a negative ssRNA virus, the spring viraemia of carp virus (SVCV), and an intracellular Gram-negative bacterial pathogen *Edwardsiella tarda*, implying that the RLR may also be able to sense elements released from bacteria. The over-expression of MDA5a and MDA5b in fish cells resulted in significant induction of type I interferon promoter activity and enabled the protection of transfected cells against SVCV infection. Furthermore, the shorter spliced form, MDA5b when co-transfected with MDA5a or mitochondrial antiviral signalling protein (MAVS), induced a significantly higher level of interferon promoter activity, indicating that MDA5b may function as an enhancer in the interaction between MDA5 and MAVS.

## Introduction

The innate immune defence against pathogenic invasion is mediated through the recognition of conserved pathogen-associated molecular patterns (PAMPs) by pattern recognition receptors (PRRs). Toll-like receptors, nucleotide-binding oligomerization domain (NOD)-like receptors and retinoic acid-inducible gene I (RIG-I)-like receptors (RLRs) are three major families of PRRs in vertebrates.^1^,^2^ The RLR family contains three members, RIG-I, melanoma differentiation-associated gene 5 (MDA5), and laboratory of genetics and physiology 2 (LGP2), which share some common and homologous core structural domains, such as caspase activation and recruitment domain (CARD) at the N terminus (with the exception of LGP2, which has no CARD), DExD/H box helicase domain (DEXDc), helicase C-terminal domain (HELICc), regulatory domain (RD; also referred to as repressor domain or C-terminal domain) at the C terminus.^3^,^4^ RIG-I signals through its tandem CARDs as a multimeric complex, and MDA5 may also signal in a homocomplex, while LGP2, through its RD at the C terminus, plays a role in inhibiting RIG-I, but not MDA5, signalling despite being able to form a complex with either RIG-I or MDA5.^6^ Both RIG-I and MDA5 initiate the host immune response through interaction of CARDs between each of the PRRs and mitochondrial antiviral signalling protein (MAVS; also known as Cardif, IPS-1 or VISA),^7^–^8^ leading to the phosphorylation of downstream signalling molecules, interferon regulatory factors 3 and 7, and to the subsequent translocation of the molecules into nucleus for inducing the expression of interferon-α/β (IFN-α/β) and IFN-stimulated genes.^1^,^7^

Among the RLR family, RIG-I was initially reported to sense viral dsRNA,^10^ and MDA5 was found also in the recognition of viral dsRNA.^5^,^11^ The recognition of dsRNA by RIG-I or MDA5 is related to different features of ligands, such as 5′-triphosphate constituents,^13^ and the length of dsRNA molecules.^14^ In mammals, RIG-I and MDA5 may both functionally sense a diverse range of negative-stranded or positive-stranded RNA viruses.^15^,^16^ Recently, it was reported that RIG-I and MDA5 may also sense bacterial RNA or DNA released from the pathogenic bacterium *Listeria monocytogenes* to trigger the production of IFN-β and interleukin-1β.^18^

In teleost fish, the homologue of human MDA5 has been characterized functionally only in rainbow trout, *Oncorhynchus mykiss*, which was responsive to the infection of RNA viruses, such as viral haemorrhagic septicaemia virus and salmon alphavirus.^19^ In the present study, the MDA5, and its shorter splicing variants, named as MDA5a and MDA5b, were identified in zebrafish, *Danio rerio*. The expression of the two variants was then examined in cell lines following infection with spring viraemia of carp virus (SVCV), a negative ssRNA virus as well as *Edwardsiella tarda*, an intracellular Gram-negative pathogenic bacterium. The role of MDA5a and MDA5b in inducing IFN promoter, and in inducing an antiviral state was also examined in a fish cell line. The possible interaction between the two variants, MDA5a and MDA5b, and between the variants and MAVS, the adaptor molecule triggered by MDA5 and RIG-I, was also examined in cell lines. The present study therefore provides the first report on the existence of MDA5 spliced variants and their functions in fish and probably in any other vertebrates.

## Materials and methods

### Cell lines and transfection

Zebrafish embryonic fibroblast (ZF4) cells^20^ were cultured at 28° in a mixture of 90% 1 : 1 mixed Dulbecco’s modified Eagle’s medium (DMEM) and Ham F12 medium (Invitrogen-Gibco, Carlsbad, CA) supplemented with 10% fetal bovine serum (Invitrogen-Gibco) according to the previous report.^21^ Epithelioma papulosum cyprini (EPC) and human embryonic kidney (HEK 293T) cells were maintained in DMEM supplemented with 5% CO_2_, 10% fetal bovine serum at 25° or 37°, respectively.

Transfection of plasmids in ZF4 cells was performed using the Amaxa Nucleofector II transfection system (Lonza, Cologne, Germany) under Program T20, whereas in EPC cells and HEK 293T cells Lipofectamine 2000 (Invitrogen) was used. All of the procedures were performed according to the manufacturers’ instructions. All experiments involving cell lines and animals complied with the local and institutional guidelines for the experimental use of animals.

### Virus and bacterium used for infection

The negative ssRNA virus SVCV was propagated in EPC cells at 25°, with titres determined by plaque assay under methylcellulose overlay (0·5% in DMEM).

*Edwardsiella tarda* PPD130/91, an intracellular Gram-negative bacterial pathogen,^22^ was grown in tryptic soy broth (BD Biosciences, Sparks, MD) liquid culture overnight at 28°. Quantification of the bacterial culture was performed by spectrophotometry and plating dilution on tryptic soy agar (BD Biosciences). SVCV and *E. tarda* were both confirmed as infective in zebrafish and ZF4 cells.^22^–^23^.

### RNA extraction, molecular cloning and plasmid construction

Total RNA was extracted using Trizol Reagent (Gibco) from organ/tissue samples, including kidney, spleen and intestine of zebrafish adults stimulated for 24 hr with the injection of 100 ng poly I:C, and the RNA was treated with RNase-free DNase I (Fermentas Life Sciences, Vilnius, Lithuania) and RiboLock™ RNase inhibitor (Fermentas Life Sciences) according to the manufacturer’s protocol. The DNase I-treated RNA was used for the synthesis of first-strand cDNA for 5′ RACE and 3′ RACE using GeneRacer™ Kit (Invitrogen). Based on the partial sequence of zebrafish MDA5 (XM_689032.3), nested primers zfMDA5-5-R1, zfMDA5-5-R2, zfMDA5-3-F1 and zfMDA5-3-F2 (Table 1) were designed and used in 5′ RACE or 3′ RACE using a GeneRacer™ Kit (Invitrogen) according to the manufacturer’s instructions. For 5′ RACE, the annealing temperature of the first round and nested PCR was 56°, whereas it was 60° and 58°, respectively, for 3′ RACE. The PCR products were cloned into the pMD18-T vector (Takara, Dalian, China) for sequencing by Sangon Biotech Co., Ltd (Shanghai, China).

**Table 1:** Primer sequences used in this study

Primer name	Sequence	Application
GeneRacer™ 5′ Primer	CGACTGGAGCACGAGGACACTGA	The first run PCR for 5′RACE
GeneRacer™ 5′ Nested Primer	GGACACTGACATGGACTGAAGGAGTA	The nested PCR for 5′RACE
zfMDA5-5-R1	TTGAGGAGCAGACGAGCACCATT	The first run PCR for 5′RACE
zfMDA5-5-R2	CTGTCGTGTTCGGCTTCTTCATCT	The nested PCR for 5′RACE
GeneRacer™ 3′ Primer	GCTGTCAACGATACGCTACGTAACG	The first run PCR for 3′RACE
GeneRacer™ 3′ Nested Primer	CGCTACGTAACGGCATGACAGTG	The nested PCR for 3′RACE
zfMDA5-3-F1	TCATAGTCCGAGAGAATGCGTCA	The first run PCR for 3′RACE
zfMDA5-3-F2	GTCACATACGGCTCCAAGAAGAA	The nested PCR for 3′RACE
pTurbo-MDA5a-F	GACCTCGAGATGGATCCAAACATGAGCAG	pTurbo-MDA5a-GFP
pTurbo-MDA5a-R	GTTGTCGACCAGTTAGTGTCCATATCTTCATC	
pTurbo-MDA5b-F	GACCTCGAGATGGATCCAAACATGAGCAG	pTurbo-MDA5b-GFP
pTurbo-MDA5b-R	GTTGTCGACTGACACTCTCTCGCCTTCTC	
pcDNA3.1-MDA5a-F	GACCTCGAGCGATGGATCCAAACATGAGCAG	pcDNA3.1-MDA5a
pcDNA3.1-MDA5a-R	CGGGGTACCGTTAGTGTCCATATCTTCATC	
pcDNA3.1-MDA5b-F	GACCTCGAGCGATGGATCCAAACATGAGCAG	pcDNA3.1-MDA5b
pcDNA3.1-MDA5b-R	CGGGGTACCACACTCTCTCGCCTTCTCTCT	
p3xFLAG-MAVS-F	CGGGGTACCAATGGCTTCACTGACACGTG	p3xFLAG-MAVS
p3xFLAG-MAVS-R	CACGGATCCATGATTGAGCTTCCAGGC	
qMDA5a-F	CCTGACGAGGAAGGCAACATTACA	Quantitative real-time PCR
qMDA5a-R	AACTGGCTTGGACTCCCACTTCAT	
qMDA5b-F	CCAGAGAGAAGGCGAGAGAGTGTTA	Quantitative real-time PCR
qMDA5b-R	TCTTCTCCAGAGCACACAAACACA	
qMAVS-F	CAGAACAACTCAGGCGACAACA	Quantitative real-time PCR
qMAVS-R	TCCTCCTCAGGCTGGTTATTAGTC	
qMx-F	AGACCATCCTCATTTCAGCAAACTCT	Quantitative real-time PCR
qMx-R	CAATCTTTTTGTTGAATGAATCCCCTG	
qGAPDH-F	GTAACTCCGCAGAAAAGCCAGAC	Quantitative real-time PCR
qGAPDH-R	CAAAAGAAACTAACACACACACA	

GeneRacer™ 5′ Primer, GeneRacer™ 5′ Nested Primer, GeneRacer™ 3′ Primer and GeneRacer™ 3′ Nested Primer are from the GeneRacer™ Kit (Invitrogen); and qGAPDH-F and qGAPDH-R were reported in a previous study.^26^

For eukaryotic expression, the open reading frame (ORF) of zebrafish MDA5a and MDA5b was cloned and inserted into the pcDNA3.1/myc-His(-)A vector (Invitrogen). For subcellular localization, the entire ORF of MDA5a and MDA5b was also inserted into the pTurboGFP-N vector (Evrogen, Moscow, Russia). The ORF of zebrafish MAVS was sub-cloned into pcDNA3.1/myc-His(-)A vector (Invitrogen) and p3xFLAG-CMV™-14 expression vector (Sigma Aldrich, St Louis, MO), respectively. All plasmid constructs were verified by sequencing analysis. The primers with the restricted enzyme cutting sites are listed in Table 1.

### Sequence analysis

Protein prediction was performed using software at the ExPASy Bioinformatics Resource Portal (http://web.expasy.org/proteomics). Analysis of conserved domains was performed using NCBI CDD.^24^ The zebrafish MDA5 cDNA sequences obtained were searched against zebrafish genome (Zv9) for further identification. The intron/exon structures of genomic sequences were determined by alignment of the full-length cDNA sequence to genomic sequence using BLAST.

### Quantitative real-time PCR

For SVCV or *E. tarda* infection, ZF4 cells were passaged into six-well plates for 24 hr at a concentration of 5 × 10^6^ cells per well. Cells were then infected with SVCV or *E. tarda* at a multiplicity of infection (MOI) of 5 or 10, respectively, and 6 and 24 hr post infection (hpi) cells were collected for RNA extraction as described above. The first-strand cDNAs were synthesized by Superscript reverse transcriptase (Fermentas Life Sciences) and oligo(dT) primer using RNase-free DNase I-treated RNA. Quantitative real-time PCR was performed using iQ™ SYBR Green Supermix (Bio-Rad, Singapore) on a Bio-Rad CFX96 Real-Time System under the following conditions: 3 min at 95°, followed by 40 cycles of 15 seconds at 94°, 15 seconds at 58° and 30 seconds at 72°. All reactions were performed in triplicate in a 96-well plate, with the mean value recorded. The relative expression level of target genes was normalized to the expression of glyceraldehyde-3-phosphate dehydrogenase (GAPDH). Fold changes were calculated by comparison with the corresponding controls using the comparative Ct method (2^−ΔΔCt^).^25^ Three experiments were conducted for statistical analysis using paired Student’s *t*-test, with the probability level of *P *<* *0·05 set as statistical significance. All primers used for quantitative real-time PCR are listed in Table 1, with one pair of primers named as qGAPDH-F/R from a previous report.^26^

### Fluorescence microscopy and luciferase activity assay

For subcellular localization of MDA5a and MDA5b, ZF4 cells were cultured in fresh flasks for 24 hr, and 5 μg of each of the following constructs, pTurbo-MDA5a-GFP, pTurbo-MDA5b-GFP and pTurboGFP-N (vector control), were transfected into 5 × 10^6^ cells using Amaxa Nucleofector II transfection system (Lonza) under Program T20. Cells were then transferred into 25-cm^2^ fresh flasks for 24 hr of culture at 28°, and transfected cells were stained with Hoechst 33342 (Sigma-Aldrich) before being examined under a fluorescence microscope (Zeiss, Oberkochen, Germany). Subsequently, cells were collected to confirm MDA5a-GFP and MDA5b-GFP fusion proteins by Western blotting, with Anti-TurboGFP antibody (Evrogen, CAT. #AB513) diluted at 1 : 5000 as the primary antibody. The bands were detected by using Immobilon™ Western Chemiluminescent HRP Substrate (Millipore Corporation, Billerica, MA) and ECL Western blot system (LAS-4000 mini; Fujifilm, Tokyo, Japan) according to the manufacturers’ instructions.

For luciferase activity assay, EPC cells seeded overnight in 24-well plates at 2 × 10^5^ cells per well were transiently transfected with 100 ng IFNpro-Luc, a zebrafish type I IFN promoter-driving luciferase plasmid^27^ and 10 ng pRL-TK (Promega, Madison, WI), together with pcDNA3.1-MDA5a, pcDNA3.1-MDA5b or pcDNA3.1 (vector control) at various concentrations (10, 50, 100 or 200 ng) using Lipofectamine 2000 (Invitrogen). Twenty-four hours post-transfection, cells were harvested and lysed using Dual-Luciferase Reporter System (Promega), with luciferase activity measured on a Junior LB9509 luminometer (Berthold, Pforzheim, Germany). Data were normalized to the Renilla Luciferase activity, and expressed as the fold change relative to control group transfected with empty vector pcDNA3.1. Cell lysates were examined by Western blotting analysis.

To determine the possible association between MDA5a, MDA5b and MAVS, EPC cells seeded overnight in 24-well plates were transiently transfected with 100 ng each of the plasmids pcDNA3.1-MDA5a, pcDNA3.1-MDA5b, or pcDNA3.1-MAVS, or with combinations of any two, together with luciferase reporter plasmids (100 ng IFNpro-Luc and 10 ng pRL-TK). The pcDNA3.1 empty vector was used to balance the total volume of transfected plasmids. Twenty-four hours post-transfection, cells were harvested and luciferase activity was measured as described above.

### Antiviral effect

EPC cells seeded in 24-well plates at a concentration of 1 × 10^6^ cells per well were transfected with 1 μg pcDNA3.1-MDA5a, pcDNA3.1-MDA5b or pcDNA3.1 empty vector (control), respectively. Twenty-four hours post-transfection, transfected cells were washed and infected with SVCV at an MOI of 1, 0·1 or 0·01, respectively, and 48 hpi, the culture supernatants were collected for the determination of virus titres by standard plaque assay. Cell monolayers were then fixed in 10% paraformaldehyde for 1 hr, before being stained with 0·5% crystal violet for the observation of cytopathic effect.

### Antibodies, co-immunoprecipitation and Western blotting

Rabbit polyclonal antibody against zebrafish MDA5 was generated by a commercial supplier (GenScript, Nanjing, China) using standard procedures with synthetic peptide, VRNRPPEPDEEAEHC of the MDA5. Mouse monoclonal antibody against Flag was purchased from Sigma.

For co-immunoprecipitation experiments, HEK 293T cells seeded in six-well plates were co-transfected with p3xFLAG-MAVS and pcDNA3.1-MDA5a, or with p3xFLAG-MAVS and pcDNA3.1-MDA5b, and with p3xFLAG-MAVS and pcDNA3.1 as control. Twenty-four hours post-transfection, cells were collected and lysed in 500 μl extraction buffer as described above. Cellular debris was removed by centrifugation at 12000 ***g*** for 10 min at 4°, and supernatants were transferred to fresh tubes and incubated overnight at 4° with anti-Flag resin conjugate (FLAG Tagged Protein Immunoprecipitation Kit; Sigma). The resin was washed six times with ice-cold wash solution as described by the manufacturer, and eluted with 40 μl 2 × SDS sample buffer by boiling for 10 min at 95°. The precipitates were detected by Western blotting with anti-MDA5 and anti-Flag antibodies.

For Western blotting, equal amounts of protein samples were separated on 8% or 12% SDS–PAGE gels and then electrophoretically transferred to a polyvinylidene difluoride membrane (Millipore Corporation). Afterwards, the membrane was blocked with PBS containing 5% non-fat dry milk for 1 hr at room temperature, before three washes, each for 5 min, in PBS containing 0·1% Tween 20 (PBST). The membrane was incubated with primary antibody in PBS containing 2% milk for 2 hr at room temperature, and 1 hr incubation with secondary antibody also at room temperature, with three washes in PBST following each incubation. The membrane was stained with Immobilon™ Western Chemiluminescent HRP Substrate (Millipore Corporation) and examined under the ECL Western blot system LAS-4000 mini; Fujifilm).

## Results

### Sequence analysis of MDA5 transcripts in zebrafish

**Figure 1:**
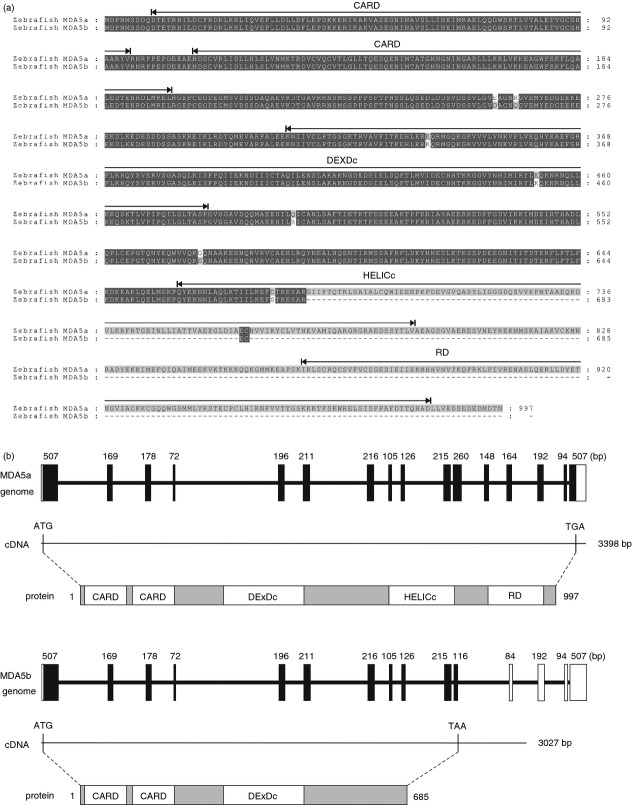
Sequence analysis of melanoma differentiation-associated gene 5 (MDA5) splicing variants in zebrafish. (a) Amino acid sequence alignment of MDA5a and MDA5b. Domains of CARD (caspase activation and recruitment domain), DEXDc (DExD/H box helicase domain), HELICc (helicase C-terminal domain) and RD (regulatory domain) are indicated by arrows. (b) Exon–intron structural analysis of zebrafish MDA5 splicing variants. Exons are shown as black boxes, with the length indicated in base pairs. The constitution of each exon corresponding to the translated protein is indicated, with the grey shaded region indicating the open reading frame. Domains in the translated protein, such as CARD, DEXDc, HELICc and RD are also shown.

Using primers to amplify the entire ORF of MDA5 in zebrafish, two transcripts named as MDA5a (GenBank accession no. JX462556) and MDA5b (JX462557), were identified, which were 3 398, and 3 027 bp encoding 997 and 685 amino acids, respectively. Using NCBI CDD (http://www.ncbi.nlm.nih.gov/Structure/cdd/docs/cdd_search.html), it was found that MDA5a and MDA5b contained conserved domains, such as two CARDs, and a DEXDc domain, with the former having an HELICc domain and an RD domain that were absent in MDA5b (Fig. 1a).

Searching the zebrafish genome, MDA5a and MDA5b were found to be encoded by a single gene, which was located on zebrafish chromosome 9. The MDA5a gene consists of 16 exons, with 15 intervening introns, spanning approximately 69 kb of genomic sequence, while MDA5b lacks partial exons 11 and 13 and the entire exon 12, so causing a premature stop in exon 11 with the transcription of a predicted 685 amoino acids, being a shorter form of MDA5 protein, named as MDA5b (Fig. 1b).

### Localization of MDA5a and MDA5b in ZF4 cells

**Figure 2:**
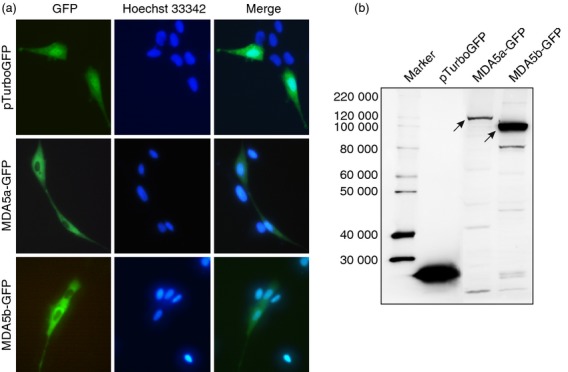
Subcellular localization of zebrafish melanoma differentiation-associated gene 5a (MDA5a) and MDA5b in ZF4 cells. (a) ZF4 cells were transiently transfected with pTurboGFP, pTurbo-MDA5a-GFP or pTurbo-MDA5b-GFP. Twenty-four hours post-transfection, cells were stained with Hoechst 33342 and photographed under a fluorescence microscope. (b) The expression of GFP fusion proteins was also confirmed by Western blotting examination using anti-TurboGFP antibody.

To determine the subcellular localization of MDA5a and MDA5b, their ORFs were inserted into the pTurboGFP-N expression vector for transient transfection in ZF4 cells. Fluorescent microscopy revealed that both pTurbo-MDA5a-GFP and pTurbo-MDA5b-GFP fusion proteins had an entire cytosolic distribution except in the nucleus, whereas control cells transfected with pTurboGFP-N empty vector showed a cytosolic as well as nucleic distribution (Fig. 2a). The presence of pTurbo-MDA5a-GFP and pTurbo-MDA5b-GFP in the cytosol was also confirmed using a polyclonal antibody against the GFP protein by Western blotting analysis (Fig. 2b).

### Expression of MDA5a, MDA5b, MAVS and Mx in ZF4 cells infected with SVCV and *E. tarda*

**Figure 3:**
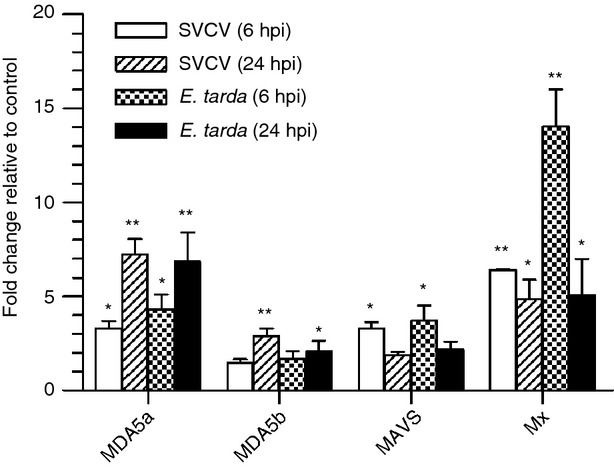
Expression analysis of zebrafish melanoma differentiation-associated gene 5a (MDA5a), MDA5b, mitochondrial antiviral signalling protein (MAVS) and Mx in ZF4 cells during spring viraemia of carp virus (SVCV) or *Edwardsiella tarda* infection. ZF4 cells seeded in six-well plates at 5 × 10^6^ cells per well were infected with SVCV (5 MOI) or *E. tarda* (10 MOI). The cells were collected at 6 and 24 hr post-infection (hpi) for RNA extraction and quantitative real-time PCR analysis. Expression level changes of target genes were calculated by normalization to GAPDH and comparison to the uninfected control cells. Data were expressed as the mean ± SD of three experiments, with the bar representing SD. *P <* *0·05, **P <* *0·01.

MDA5a and MDA5b were both significantly up-regulated in ZF4 cells in response to SVCV or to *E. tarda* infection in the 24-hr experimental period, with only one exception that MDA5b had a statistically non-significant increase at 6 hr following SVCV infection (Fig. 3). MDA5a had in general a higher level of expression, when compared with MDA5b, and its significant increase following SVCV or *E. tarda* infection was respectively above threefold and fourfold at 6 hpi, and all were about sevenfold at 24 hpi (Fig. 3). MDA5b increased about 1·5-fold at 6 hr following SVCV or *E. tarda* infection, and accordingly about threefold and twofold at 24 hpi (Fig. 3).

MAVS and Mx (myxovirus resistance A, GenBank accession no. NM_182942.4) were also up-regulated at mRNA level in ZF4 cells following SVCV or *E. tarda* infection (Fig. 3). MAVS was significantly up-regulated only at the early stage of infection, i.e. at 6 hpi, with the expression being above threefolds higher following SVCV or *E. tarda* infection; but its increased expression was not significant at 24 hpi. In addition, significantly increased expression of the antiviral protein Mx, which is known to be up-regulated in virus infection, was also observed in ZF4 cells following either SVCV or *E. tarda* infection, with a rather higher expression level (about 14-fold) observed at 6 hr following *E. tarda* infection (Fig. 3).

### Luciferase assay on the activation of IFN promoter by MDA5a and MDA5b

**Figure 4:**
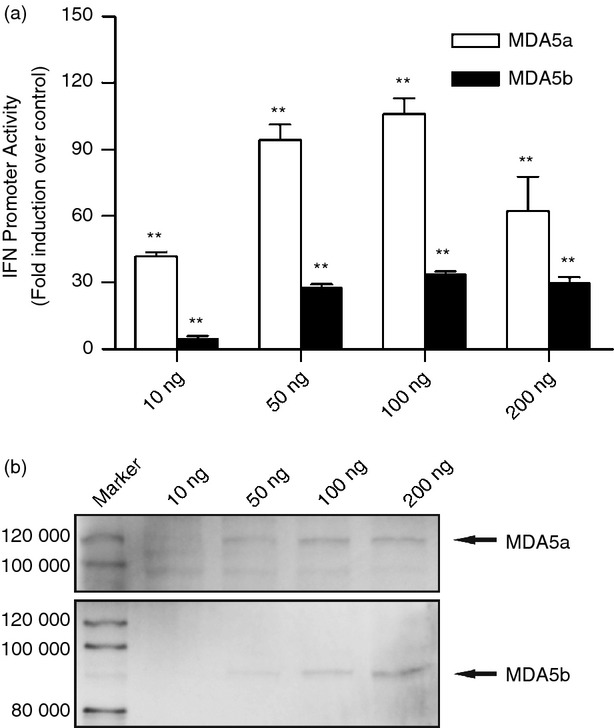
Luciferase assay on the induction of interferon (IFN) promoter activity by melanoma differentiation-associated gene 5a (MDA5a) and MDA5b in zebrafish. (a) Detection of luciferase activity at 24 hr post transfection in epithelioma papulosum cyprini (EPC) cells which were seeded in 24-well plates for being transfected with 100 ng IFNpro-Luc, 10 ng pRL-TK together with indicated amounts of pcDNA3.1-MDA5a, pcDNA3.1-MDA5b or pcDNA3.1 (vector control). (b) Lysates of cells transfected with pcDNA3.1-MDA5a or pcDNA3.1-MDA5b plasmids were analysed for MDA5a or MDA5b expression by Western blotting assays using rabbit polyclonal anti-MDA5 antibody. Bars represent SD obtained by measuring each sample in triplicate. *P <* *0·05, **P <* *0·01.

Subsequently, luciferase assay was used to determine whether MDA5a and MDA5b induced the activation of IFN, with the constructed expression plasmids, pcDNA3.1-MDA5a and pcDNA3.1-MDA5b co-transfected with IFN reporter plasmid in EPC cells. The over-expression of MDA5a provoked a strong activation of IFN promoter, by up to 42-fold against the control when the amount of each plasmid was 10 ng, and up to 106-fold when the amount was 100 ng. MDA5b also induced the activation of IFN promoter, although the induced level was not as high as that for MDA5a, with fivefold when the plasmid amount was 10 ng and 34-fold for 100 ng (Fig. 4a). Additionally, the protein of the transfected EPC cells was also examined by Western blotting analysis using polyclonal antibody against zebrafish MDA5, which confirmed the successful over-expression of MDA5a and MDA5b in EPC cells after transfection (Fig. 4b).

### Antiviral effect of MDA5a and MDA5b in transfected cell line

**Figure 5:**
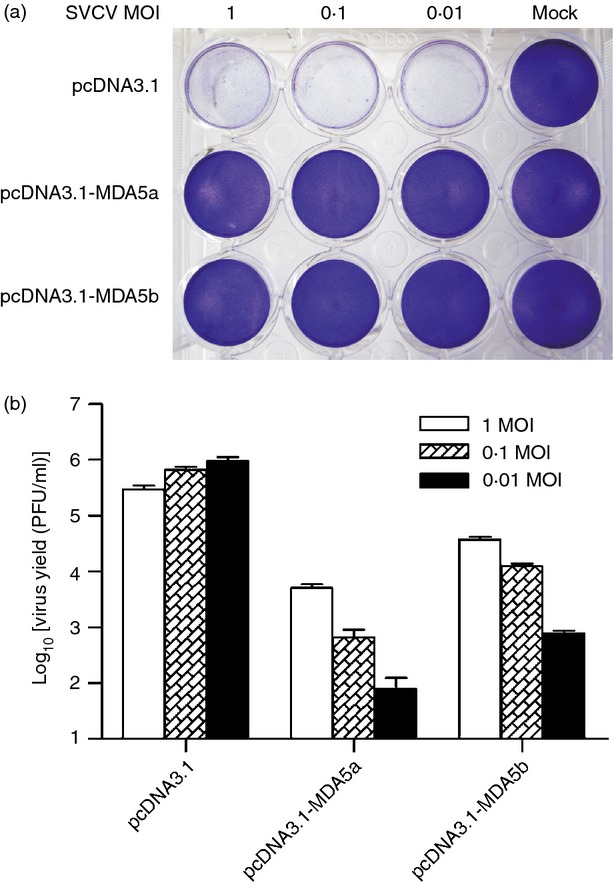
Antiviral assay on zebrafish melanoma differentiation-associated gene 5a (MDA5a) and MDA5b expressed in fish cell line. Epithelioma papulosum cyprini (EPC) cells seeded in 24-well plates were transfected with 1 μg pcDNA3.1-MDA5a, or pcDNA3.1-MDA5b, or empty vector (pcDNA3.1) as control. Twenty-four hours post-transfection, cells were infected with spring viraemia of carp virus (SVCV) at various MOI (1, 0·1 and 0·01). (a) 48 hr post-infection, the culture supernatants were collected and the cell monolayers were fixed with 10% paraformaldehyde and stained with 0·5% crystal violet. (b) The viral titres of the supernatants were determined by plaque assays on EPC cells. Data are expressed as mean ± SD of three experiments.

The antiviral role of the two variants was examined in EPC cells transfected with pcDNA3.1-MDA5a, or pcDNA3.1-MDA5b or pcDNA3.1 empty vector as control, followed by infection with SVCV at various MOI (1, 0·1, 0·01). Complete cytopathic effect was found in control cells 48 hpi, whereas the transfection of either pcDNA3.1-MDA5a or pcDNA3.1-MDA5b protected the cells against SVCV infection (Fig. 5a). Accordingly, an SVCV titre of 5·1 × 10^3^ plaque-forming units (PFU)/ml was detected in supernatants from MDA5a-overexpressing cells infected with SVCV at 1 MOI, which was 59-fold lower relative to control cells (3·0 × 10^5^ PFU/ml). Virus titres of 6·9 × 10^2^ PFU/ml and 8·3 × 10^1^ PFU/ml were detected in the supernatants from MDA5a-overexpressing cells with SVCV at 0·1 and 0·01 MOI, respectively, which were 952-fold and 11 500-fold lower relative to control cells (6·6 × 10^5^ PFU/ml at 0·1 MOI, 9·6 × 10^5^ PFU/ml at 0·01 MOI). Moreover, the detected SVCV virus titres in the supernatants from MDA5b-overexpressing cells were 3·8 × 10^4^ PFU/ml at 1 MOI, 1·2 × 10^4^ PFU/ml at 0·1 MOI and 7·8 × 10^2^ PFU/ml at 0·01 MOI, which were 7-, 53- and 1223-fold lower relative to control cells, respectively (Fig. 5b).

### Interaction of MDA5 variants and between the variants and MAVS in IFN signalling

**Figure 6:**
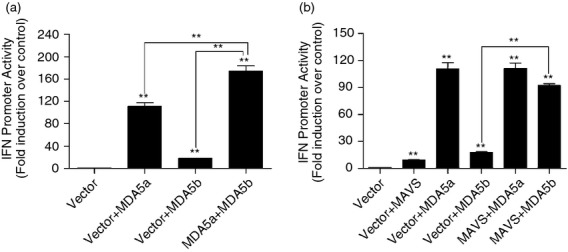
Association of zebrafish melanoma differentiation-associated gene 5a (MDA5a), MDA5b and mitochondrial antiviral signalling protein (MAVS) in the activation of interferon (IFN) promoter. Epithelioma papulosum cyprini (EPC) cells seeded in 24-well plates were co-transfected with IFNpro-Luc (100 ng), pRL-TK (10 ng) together with pcDNA3.1-MDA5a (100 ng), pcDNA3.1-MDA5b (100 ng) and pcDNA3.1-MAVS alone or in combination of two, and the pcDNA3.1 empty vector was used as control to balance the total volume of transfected plasmids. Twenty-four hours post-transfection, cells were harvested for detecting luciferase activity. (a) Association of zebrafish MDA5a and MDA5b in the induction of IFN promoter activity. (b) Association of zebrafish MDA5a, MDA5b and MAVS in the induction of IFN promoter activity. All data are expressed as mean ± SD of three experiments, with bars representing SD. *P <* *0·05, **P <* *0·01.

To reveal possible interactive roles between MDA5a and MDA5b in IFN signalling, plasmids expressing separately MDA5a or MDA5b were co-transfected into EPC cells with the IFN reporter plasmid. The luciferase assay showed that the co-transfection of MDA5a with MDA5b induced significantly higher levels of IFN promoter activity, when compared with separate over-expression of MDA5a or MDA5b (Fig. 6a). Furthermore, the co-expression of MDA5b with MAVS induced a significantly higher level of IFN activation in comparison with the only transfection of MDA5b, while co-expression of MDA5a with MAVS showed no significant impact on the IFN promoter activity when compared with the activation level in the transfection of MDA5a alone (Fig. 6b). These results suggested that the splicing variant MDA5b may act as a positive regulator of MDA5a-mediated IFN activation.

**Figure 7:**
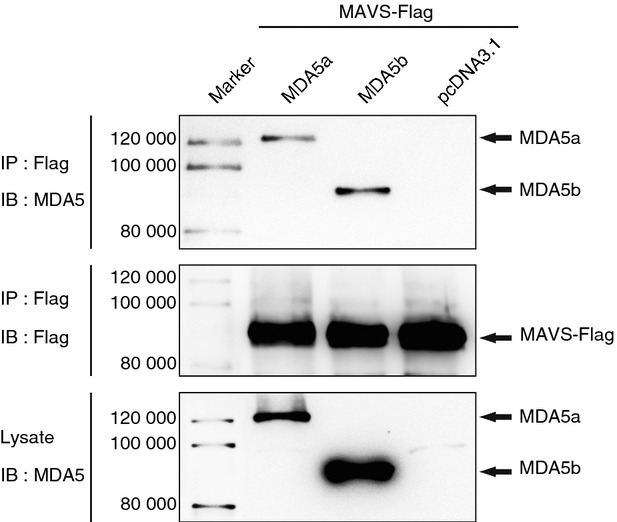
Interaction of melanoma differentiation-associated gene 5a (MDA5a) and MDA5b with mitochondrial antiviral signalling protein (MAVS) in zebrafish. HEK 293T cells seeded in six-well plates were co-transfected with 2 μg p3xFLAG-MAVS and 2 μg pcDNA3.1-MDA5a, pcDNA3.1-MDA5b or pcDNA3.1 (vector control). Twenty-four hours post-transfection, cell lysates were immunoprecipitated with anti-Flag antibody (covalently conjugated to agarose beads) and immunoblotting with anti-MDA5 antibody (upper panels), the MAVS-Flag fusion protein bound to anti-Flag-agarose beads was detected by immunoblotting with anti-Flag antibody (middle panels), and the cell lysates were also examined by immunoblotting with anti-MDA5 antibody (lower panels).

Finally, the interaction of each variant, MDA5a and MDA5b, with MAVS was determined by co-immunoprecipitation with Tag or specific antibodies. In HEK 293T cells co-transfected with p3xFLAG-MAVS and pcDNA3.1-MDA5a, anti-Flag antibody-immunoprecipitated protein complex was recognized by anti-MDA5 antibody (Fig. 7). Moreover, in cells co-transfected with p3xFLAG-MAVS and pcDNA3.1-MDA5b, co-immunoprecipitation and Western blotting analysis also showed that the MDA5b interacted with MAVS (Fig. 7). These data suggested that both MDA5a and MDA5b could form a complex with MAVS during the signal pathway.

## Discussion

In this study, MDA5 and its shorter splicing form have been identified for the first time in a teleost fish, the zebrafish, representing also the first report of MDA5 variants in vertebrates. MDA5a and MDA5b can all activate the expression of type I IFN, as demonstrated using zebrafish type I promoter-driving luciferase plasmid in a fish cell line, and when transfected, they can all protect cells against the infection of negative ssRNA virus, SVCV. Intriguingly, it is found that the MDA5a and MDA5b can all interact with their adaptor molecule, MAVS, and that the shorter form, MDA5b, functions also as an enhancer of the MDA5 signalling in the activation of IFN expression.

Research in mammals has shown that MDA5 is able to immunologically recognize positive-stranded ssRNA viruses, such as picornavirus, West Nile virus and Dengue virus, and dsRNA viruses, such as reoviruses and synthetic analogue of dsRNA virus, poly I:C.^12^–^30^ The only research on MDA5 in other classes of vertebrates was carried out in rainbow trout, and it is revealed that the expression of MDA5 can be significantly induced in cell lines following the infection of negative-stranded, or positive-stranded, ssRNA viruses, such as viral haemorrhagic septicaemia virus and salmon alphavirus, respectively, and that its over-expression can protect cells against viral haemorrhagic septicaemia virus infection.^19^ In the present study, MDA5 and its shorter splicing form, MDA5b, can be significantly induced at mRNA level in cell lines following the infection of negative-stranded ssRNA virus, SVCV, and can also protect cells against SVCV infection. Taken together, it is likely that MDA5 may be able to immunologically recognize RNA viruses, ranging from ssRNA and dsRNA viruses to negative- and positive-stranded RNA viruses. Additionally, Luthra *et al*.^31^ reported that mammalian MDA5 sense viral mRNA from parainfluenza 5 virus in association with RNase L, which cleaves viral mRNA into small RNA, and such RNA products were suggested to have cleavage marker or processing signature and could be recognized by MDA5. Pichlmair *et al*.^32^ also suggested that higher-order RNA structures of dsRNA and ssRNA contributed to the induction of MDA5-mediated IFN activation. Hence, the recognition of extensive viral RNA by MDA5 requires some conserved structures or signals that distinguish viral from host cellular RNA. However, exact features of the viral PAMPs recognized by MDA5 remain largely undefined.

In addition to viral RNA recognition for the production of type I IFN,^15^–^33^ cytosolic RLRs, RIG-I, MDA5 and STING were found to be able to recognize nucleic acids RNA/DNA secreted from infected live bacteria, *L*. *monocytogenes* with the subsequent triggering of IFN-β production.^18^ *Listeria monocytogenes* is an intracellular Gram-positive pathogen, and the secreted RNA/DNA may be mediated through SecA2, which is one of the two secretion pathways present in some Gram-positive pathogenic bacteria, and contributes to the secretion of specific virulence factors,^34^ as the mutant bacteria lacking SecA2 elicited less RIG-I and MDA5-dependent IFN-β production, although the exact feature of the RNA/DNA is unknown.^18^ The *E. tarda*, used in the present study is also intracellular, but Gram-negative, and possesses secretion systems, with types III and VI secretion systems as the main tools for the secretion of virulence factors;^35^–^36^ but it is not yet known that nucleic acids, or small RNA/DNA, are secreted through the systems, which may be of significance for further study. However, it can be at least tentatively concluded that the cytosolic RLR, MDA5, in mammals and in teleost fish can recognize elements from intracellular Gram-positive or Gram-negative bacteria.

More interestingly, a spliced shorter form, MDA5b, was found in zebrafish in addition to the presence of the mammalian homologue, MDA5. It is revealed that MDA5b resulted from the alternative splicing of the same gene as MDA5, and its expression can also be up-regulated by SVCV and *E. tarda* infection; but MDA5b lacks the HELICc and RD, which are present in MDA5. It has been demonstrated that RD in RIG-I and LGP2 plays a role in keeping the molecules in an inactive conformation, and upon ligand binding, the RD unmasks CARDs for signalling activity.^6^ However, such a role was not found in MDA5, and it the exact role of RD in MDA5 is unknown.^6^–^37^ In comparison of the structural domains of MDA5a and MDA5b, it is suggested that the N-terminal CARDs and DEXDc domains of MDA5 are sufficient to induce an antiviral response, which is consistent with the finding in RIG-I that the over-expression of the N-terminal CARDs was only able to protect cells against virus infection,^38^ although a lower level of IFN induction was observed for MDA5b. On the other hand, when co-transfected with MAVS or MDA5a, MDA5b induced much more significantly the level of IFN promoter activity. It is therefore hypothesized that in addition to its IFN-inducing role, the splicing shorter variant, MDA5b, which lacks HELICc and RD, may function as a positive regulator in MDA5-mediated induction of type I IFN production, being conserved in sensing PAMPs but diversified in exemplifying the IFN production and immune response.

Alternative splicing has been reported as a major source for producing functional diversity of proteins,^39^ and several cases have been reported for other intracellular sensors of PAMPs, or even RLRs. In mammals, a short isoform of NOD2 was identified, which served as an endogenous inhibitor of NOD2 signalling pathways.^40^–^41^ Three splicing variants of MAVS were reported in mammals and showed different roles in RIG-I/MAVS signalling, in which one splicing variant MAVS 1a functions as an inhibitor of RIG-I/MAVS-mediated nuclear factor-κB and IFN-β activation, whereas the other, MAVS 1b, activates IFN-β promoter but not nuclear factor-κB pathway.^42^ In teleost fish, LGP2 was found to have a variant, named as LGP2b, which played an inhibitory role in LGP2-mediated antiviral signalling.^19^ However, the exact mechanism involved in the amplification of IFN promoter activity by the co-transfection of MDA5b with MDA5a or MAVS requires further investigation.

In conclusion, MDA5 and its shorter splicing variant, named as MDA5a and MDA5b, respectively, were identified in zebrafish. Both MDA5a and MDA5b can be induced at mRNA level in cells infected with ssRNA virus, SVCV and intracellular Gram-negative bacteria *E. tarda*, and the RLRs can also trigger type I IFN promoter activity and an antiviral state in cell lines. Interestingly, MDA5b when co-transfected with MAVS or MDA5a, induced a significantly higher level of IFN promoter activity, although it exhibited a relatively lower level of effect on the promoter activity. It is therefore considered that MDA5b may function as an enhancer in the interaction between MDA5 and MAVS, but the interactive mechanism remains to be further investigated.
